# 947. Bed Alarm Reminder Signage: A Nurse-led Fall Prevention Strategy in Burn Trauma Care

**DOI:** 10.1093/jbcr/irag033.514

**Published:** 2026-04-06

**Authors:** Paula Cannavina

**Affiliations:** Warden Burn Center at Orlando Health Orlando Regional Medical Center, Orlando, FL

## Abstract

**Introduction:**

Inpatient falls lead to patient harm, are costly, and studies show that 3.6% of patients fall throughout the course of their admission. Falls disrupt their healing process and increase the patient’s length of stay exponentially. The Burn-Trauma Stepdown unit’s (BTSDU) population consists of vulnerable patients with limited mobility, increasing the risk for falls. This nurse-led project was developed to evaluate the use of visual signage as a fall prevention strategy to identify patients at risk for falls.

**Methods:**

This quality improvement project is being conducted on a 16-bed Burn Trauma Stepdown Unit. The signs were created by a charge nurse and placed on patient’s doors at eye level; ^2^the verbiage read, "Nursing staff, before exiting the room, is the bed alarm on?." Baseline fall data were collected over three months before the intervention. Following sign placement, data were collected over a subsequent three-month period.

**Results:**

Following the intervention, a clear reduction in falls was noted within the targeted population.

**Conclusions:**

The implementation of Bed Alarm Reminder Signage as a nurse-led fall prevention strategy in Burn Trauma care demonstrates a proactive and patient-centered approach to enhancing safety. By integrating visual cues and empowering nursing staff to lead the initiative, this strategy not only reinforces awareness but also promotes accountability and timely intervention. The use of nurse-designed reminder signage is thus far demonstrating improved bed alarm compliance and contributing to a reduction in patient falls in a burn trauma care setting while highlighting the effectiveness of simple, low-cost interventions in high-risk care environments.

**Applicability of Research to Practice:**

This study highlights a replicable, zero-cost, nurse-driven intervention that can be implemented across similar burn care units to enhance fall prevention strategies.

**Funding for the study:**

N/A.

